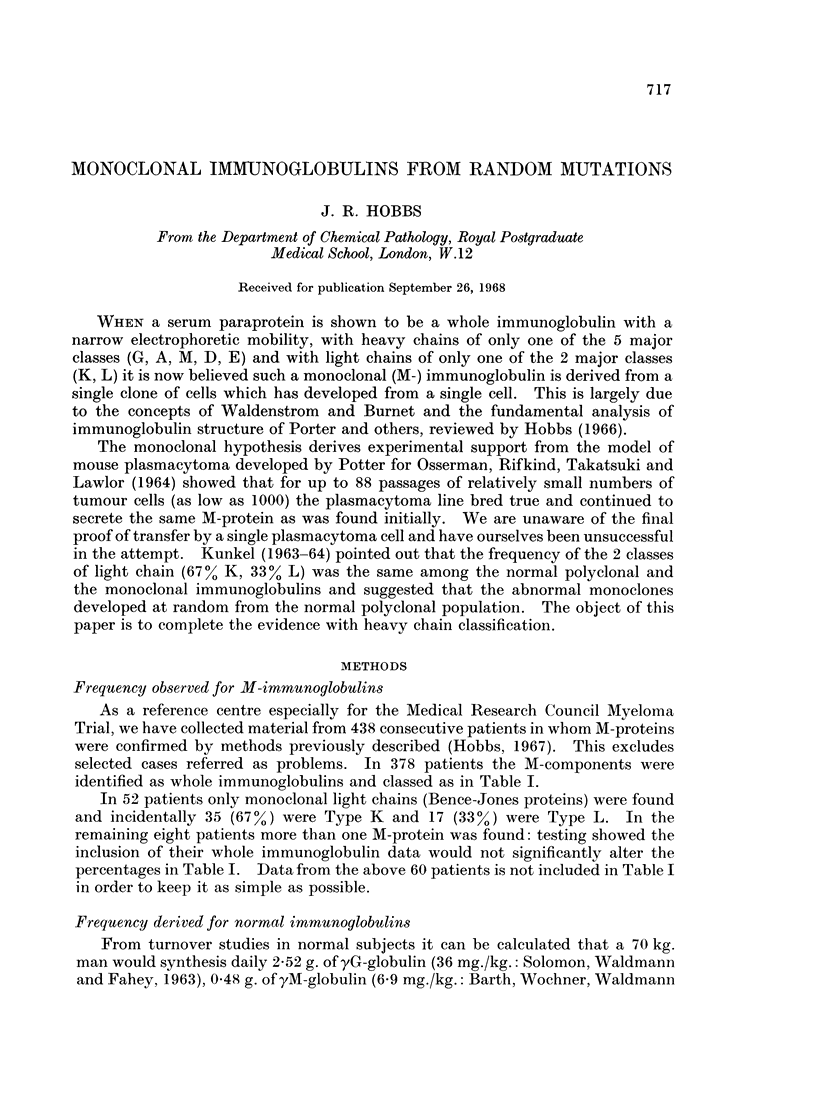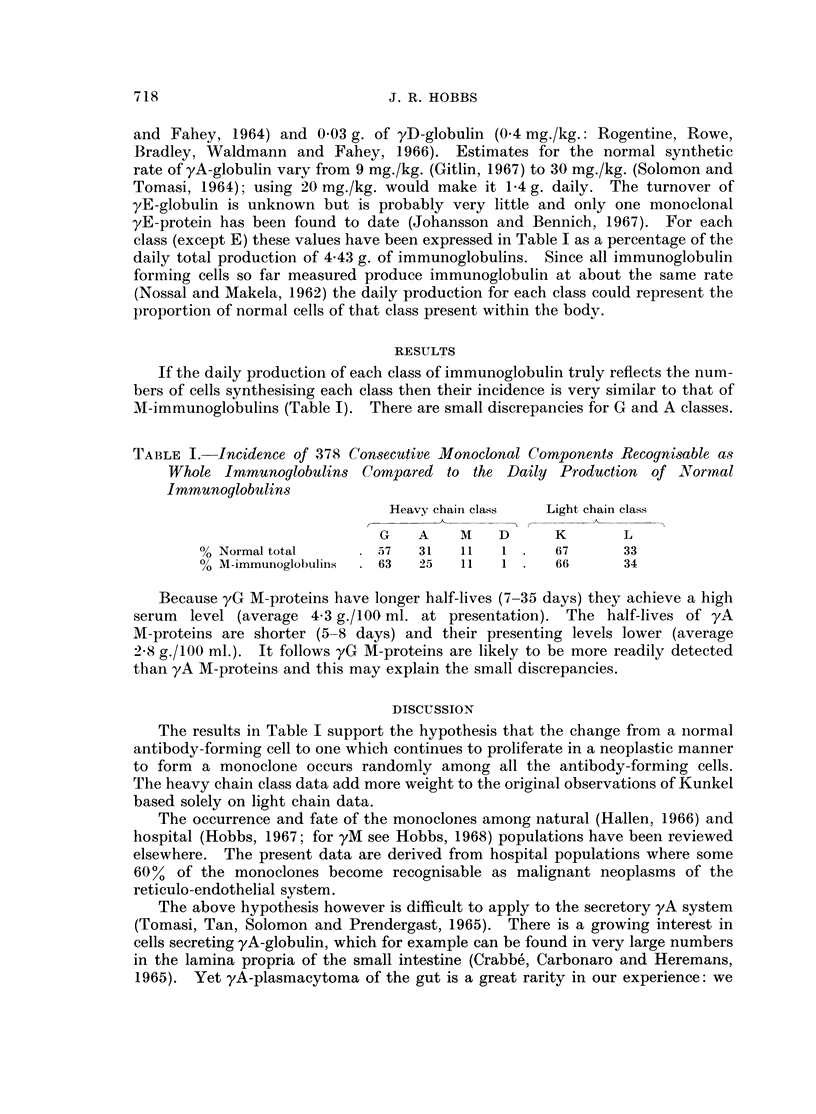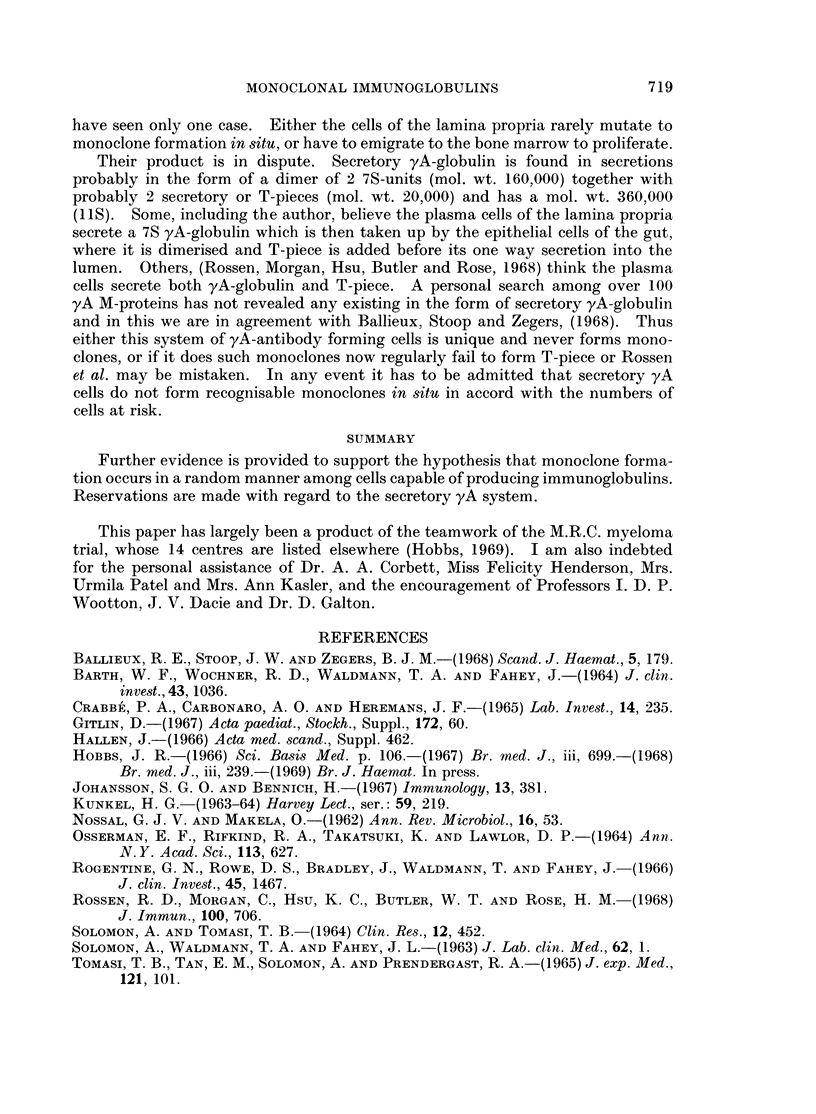# Monoclonal immunoglobulins from random mutations.

**DOI:** 10.1038/bjc.1968.85

**Published:** 1968-12

**Authors:** J. R. Hobbs


### MONOCLONAL IMMUNOGLOBULINS FROM RANDOM MUTATIONS

J. R. HOBBS

*From the Department of Chemical Pathology, Royal Postgraduate Medical School, London, W.12*

Received for publication September 26, 1968

WHEN a serum paraprotein is shown to be a whole immunoglobulin with a narrow electrophoretic mobility, with heavy chains of only one of the 5 major classes (G, A, M, D, E) and with light chains of only one of the 2 major classes (K, L) it is now believed such a monoclonal (M-) immunoglobulin is derived from a single clone of cells which has developed from a single cell. This is largely due to the concepts of Waldenstrom and Burnet and the fundamental analysis of immunoglobulin structure of Porter and others, reviewed by Hobbs (1966).

The monoclonal hypothesis derives experimental support from the model of mouse plasmacytoma developed by Potter for Osserman, Rifkind, Takatsuki and Lawlor (1964) showed that for up to 88 passages of relatively small numbers of tumour cells (as low as 1000) the plasmacytoma line bred true and continued to secrete the same M-protein as was found initially. We are unaware of the final proof of transfer by a single plasmacytoma cell and have ourselves been unsuccessful in the attempt. Kunkel (1963–64) pointed out that the frequency of the 2 classes of light chain (67% K, 33% L) was the same among the normal polyclonal and the monoclonal immunoglobulins and suggested that the abnormal monoclones developed at random from the normal polyclonal population. The object of this paper is to complete the evidence with heavy chain classification.

### METHODS

### *Frequency observed for M-immunoglobulins*

As a reference centre especially for the Medical Research Council Myeloma Trial, we have collected material from 438 consecutive patients in whom M-proteins were confirmed by methods previously described (Hobbs, 1967). This excludes selected cases referred as problems. In 378 patients the M-components were identified as whole immunoglobulins and classed as in Table I.

In 52 patients only monoclonal light chains (Bence-Jones proteins) were found and incidentally 35 (67%) were Type K and 17 (33%) were Type L. In the remaining eight patients more than one M-protein was found: testing showed the inclusion of their whole immunoglobulin data would not significantly alter the percentages in Table I. Data from the above 60 patients is not included in Table I in order to keep it as simple as possible.

### *Frequency derived for normal immunoglobulins*

From turnover studies in normal subjects it can be calculated that a 70 kg. man would synthesis daily 2·52 g. of $\gamma$G-globulin (36 mg./kg.: Solomon, Waldmann and Fahey, 1963), 0·48 g. of $\gamma$M-globulin (6·9 mg./kg.: Barth, Wochner, Waldmann and Fahey, 1964) and 0·03 g. of $\gamma$D-globulin (0·4 mg./kg.: Rogentine, Rowe, Bradley, Waldmann and Fahey, 1966). Estimates for the normal synthetic rate of $\gamma$A-globulin vary from 9 mg./kg. (Gitlin, 1967) to 30 mg./kg. (Solomon and Tomasi, 1964); using 20 mg./kg. would make it 1·4 g. daily. The turnover of $\gamma$E-globulin is unknown but is probably very little and only one monoclonal $\gamma$E-protein has been found to date (Johansson and Bennich, 1967). For each class (except E) these values have been expressed in Table I as a percentage of the daily total production of 4·43 g. of immunoglobulins. Since all immunoglobulin forming cells so far measured produce immunoglobulin at about the same rate (Nossal and Makela, 1962) the daily production for each class could represent the proportion of normal cells of that class present within the body.

### RESULTS

If the daily production of each class of immunoglobulin truly reflects the numbers of cells synthesising each class then their incidence is very similar to that of M-immunoglobulins (Table I). There are small discrepancies for G and A classes.

TABLE I.—*Incidence of* 378 *Consecutive Monoclonal Components Recognisable as Whole Immunoglobulins Compared to the Daily Production of Normal Immunoglobulins*

| | Heavy chain class | | | | Light chain class | |
|---|---|---|---|---|---|---|
| | G | A | M | D | K | L |
| % Normal total . | 57 | 31 | 11 | 1 . | 67 | 33 |
| % M-immunoglobulins . | 63 | 25 | 11 | 1 . | 66 | 34 |

Because $\gamma$G M-proteins have longer half-lives (7–35 days) they achieve a high serum level (average 4·3 g./100 ml. at presentation). The half-lives of $\gamma$A M-proteins are shorter (5–8 days) and their presenting levels lower (average 2·8 g./100 ml.). It follows $\gamma$G M-proteins are likely to be more readily detected than $\gamma$A M-proteins and this may explain the small discrepancies.

### DISCUSSION

The results in Table I support the hypothesis that the change from a normal antibody-forming cell to one which continues to proliferate in a neoplastic manner to form a monoclone occurs randomly among all the antibody-forming cells. The heavy chain class data add more weight to the original observations of Kunkel based solely on light chain data.

The occurrence and fate of the monoclones among natural (Hallen, 1966) and hospital (Hobbs, 1967; for $\gamma$M see Hobbs, 1968) populations have been reviewed elsewhere. The present data are derived from hospital populations where some 60% of the monoclones become recognisable as malignant neoplasms of the reticulo-endothelial system.

The above hypothesis however is difficult to apply to the secretory $\gamma$A system (Tomasi, Tan, Solomon and Prendergast, 1965). There is a growing interest in cells secreting $\gamma$A-globulin, which for example can be found in very large numbers in the lamina propria of the small intestine (Crabbé, Carbonaro and Heremans, 1965). Yet $\gamma$A-plasmacytoma of the gut is a great rarity in our experience: we have seen only one case. Either the cells of the lamina propria rarely mutate to monoclone formation *in situ*, or have to emigrate to the bone marrow to proliferate.

Their product is in dispute. Secretory $\gamma$A-globulin is found in secretions probably in the form of a dimer of 2 7S-units (mol. wt. 160,000) together with probably 2 secretory or T-pieces (mol. wt. 20,000) and has a mol. wt. 360,000 (11S). Some, including the author, believe the plasma cells of the lamina propria secrete a 7S $\gamma$A-globulin which is then taken up by the epithelial cells of the gut, where it is dimerised and T-piece is added before its one way secretion into the lumen. Others, (Rossen, Morgan, Hsu, Butler and Rose, 1968) think the plasma cells secrete both $\gamma$A-globulin and T-piece. A personal search among over 100 $\gamma$A M-proteins has not revealed any existing in the form of secretory $\gamma$A-globulin and in this we are in agreement with Ballieux, Stoop and Zegers, (1968). Thus either this system of $\gamma$A-antibody forming cells is unique and never forms monoclones, or if it does such monoclones now regularly fail to form T-piece or Rossen *et al.* may be mistaken. In any event it has to be admitted that secretory $\gamma$A cells do not form recognisable monoclones *in situ* in accord with the numbers of cells at risk.

### SUMMARY

Further evidence is provided to support the hypothesis that monoclone formation occurs in a random manner among cells capable of producing immunoglobulins. Reservations are made with regard to the secretory $\gamma$A system.

This paper has largely been a product of the teamwork of the M.R.C. myeloma trial, whose 14 centres are listed elsewhere (Hobbs, 1969). I am also indebted for the personal assistance of Dr. A. A. Corbett, Miss Felicity Henderson, Mrs. Urmila Patel and Mrs. Ann Kasler, and the encouragement of Professors I. D. P. Wootton, J. V. Dacie and Dr. D. Galton.

### REFERENCES

Ballieux, R. E., Stoop, J. W. and Zegers, B. J. M.—(1968) *Scand. J. Haemat.*, **5**, 179.

Barth, W. F., Wochner, R. D., Waldmann, T. A. and Fahey, J.—(1964) *J. clin. invest.*, **43**, 1036.

Crabbé, P. A., Carbonaro, A. O. and Heremans, J. F.—(1965) *Lab. Invest.*, **14**, 235.

Gitlin, D.—(1967) *Acta paediat., Stockh.*, Suppl., **172**, 60.

Hallen, J.—(1966) *Acta med. scand.*, Suppl. 462.

Hobbs, J. R.—(1966) *Sci. Basis Med.* p. 106.—(1967) *Br. med. J.*, iii, 699.—(1968) *Br. med. J.*, iii, 239.—(1969) *Br. J. Haemat.* In press.

Johansson, S. G. O. and Bennich, H.—(1967) *Immunology*, **13**, 381.

Kunkel, H. G.—(1963–64) *Harvey Lect.*, ser.: **59**, 219.

Nossal, G. J. V. and Makela, O.—(1962) *Ann. Rev. Microbiol.*, **16**, 53.

Osserman, E. F., Rifkind, R. A., Takatsuki, K. and Lawlor, D. P.—(1964) *Ann. N.Y. Acad. Sci.*, **113**, 627.

Rogentine, G. N., Rowe, D. S., Bradley, J., Waldmann, T. and Fahey, J.—(1966) *J. clin. Invest.*, **45**, 1467.

Rossen, R. D., Morgan, C., Hsu, K. C., Butler, W. T. and Rose, H. M.—(1968) *J. Immun.*, **100**, 706.

Solomon, A. and Tomasi, T. B.—(1964) *Clin. Res.*, **12**, 452.

Solomon, A., Waldmann, T. A. and Fahey, J. L.—(1963) *J. Lab. clin. Med.*, **62**, 1.

Tomasi, T. B., Tan, E. M., Solomon, A. and Prendergast, R. A.—(1965) *J. exp. Med.*, **121**, 101.